# Predicting the Spatial-Temporal Distribution of Human Brucellosis in Europe Based on Convolutional Long Short-Term Memory Network

**DOI:** 10.1155/2022/7658880

**Published:** 2022-08-03

**Authors:** Li Shen, Chenghao Jiang, Minghao Sun, Xuan Qiu, Jiaqi Qian, Shuxuan Song, Qingwu Hu, Heilili Yelixiati, Kun Liu

**Affiliations:** ^1^School of Remote Sensing and Information Engineering, Wuhan University, Wuhan, China; ^2^Department of Epidemiology, Ministry of Education Key Lab of Hazard Assessment and Control in Special Operational Environment, School of Public Health, Air Force Medical University, Xi'an, China

## Abstract

Brucellosis is a chronic infectious disease caused by brucellae or other bacteria directly invading human body. Brucellosis presents the aggregation characteristics and periodic law of infectious diseases in temporal and spatial distribution. Taking major European countries as an example, this study established the temporal and spatial distribution sequence of brucellosis, analyzed the temporal and spatial distribution characteristics of brucellosis, and quantitatively predicted its epidemic law by using different traditional or machine learning models. This paper indicates that the epidemic of brucellosis in major European countries has statistical periodic characteristics, and in the same cycle, brucellosis has the characteristics of piecewise trend. Through the comparison of the prediction results of the three models, it is found that the prediction effect of long short-term memory and convolutional long short-term memory models is better than autoregressive integrated moving average model. The first mock exam using Conv layer and data vectorizations predicted that the convolutional long short-term memory model outperformed the traditional long short-term memory model. Compared with the monthly scale, the prediction of the trend stage of brucellosis can achieve better results under the single model prediction. These findings will help understand the development trend and liquidity characteristics of brucellosis, provide corresponding scientific basis and decision support for potential risk assessment and brucellosis epidemic prevention and control, and reduce the loss of life and property.

## 1. Introduction

Brucellosis is a chronic infectious disease caused by brucellae bacteria invading human body. The main clinical symptoms include fever, fatigue, muscle, and joint pain [1]. With the development of global animal husbandry, brucellosis has become one of the important public health problems in the world. People suffering from *Brucella* bacteria often have repeated attacks if they cannot see a doctor in time or are misdiagnosed. In a long term, it will influence economy of the region, even the world.

At present, there are many studies on the epidemic trend and influencing factors of brucellosis in the world. Through spatial autocorrelation analysis, Wu et al. found that brucellosis high aggregation areas in Zhejiang Province spread year by year. With the increasing of animal husbandry production, the number of brucellosis cases is also rising [2]. Silva et al. used a generalized additive model to detect high-risk areas of brucellosis in Mato Grosso State, Brazil [3]. Peng et al. found that the epidemic scope of brucellosis in China has been expanding in recent years. Using multiple linear regression analysis, they concluded that the amount of sheep stock, GDP, and some meteorological factors were significantly correlated with brucellosis incidence rate [4]. In addition to conventional statistical data analysis, Wang et al. used artificial neural network model and remote sensing satellite data to explore the impact of natural environmental variables on brucellosis, such as vegetation and climate [5]. Ducrotoy et al. found that, under the background of the increasing level of agricultural intensification, the large-scale centralized breeding of animal husbandry will increase the risk of zoonotic infection, including brucellosis [6].

Overall, the current study considers that the incidence rate and spatial and temporal distribution of brucellosis include two aspects, including natural environment and social economy, but mainly related to the development of social economy and agriculture and animal husbandry [7, 8].

On the other hand, there are still some deficiencies in the global research on *Brucella*. At present, the research on the distribution of brucellosis in the intercontinental range is mainly aimed at the long-term time evolution and biological immunology, but the real-time research is relatively few [9, 10]. Moreover, there is no relevant research on the prediction of brucellosis based on machine learning method at home and abroad, and the distribution and prediction based on spatial analysis also wait for further improvement.

In this paper, the ConvLSTM network based on spatial grid is used to predict brucellosis and panel analysis, so as to explore the development trend and fluidity characteristics of brucellosis. The conclusions of the study will provide corresponding scientific basis and decision support for potential risk assessment and brucellosis epidemic prevention and control and reduce the loss of life and property.

## 2. Data Preparation

### 2.1. Study Area

Brucellosis is prevalent worldwide, especially in areas with developed animal husbandry. Although some countries in Northern and Central Europe, like Sweden, Austria, and Belgium, have been granted brucellosis-free status, the Mediterranean basin remains with high prevalence of brucellosis and sporadic cases have been reported in Greece and Portugal in recent years [11]. The overall incidence of human brucellosis in Europe peaked in 2016, which was associated with the high proportion of *Brucella*‐infected cattle and small ruminant populations in Italy, Greece, and Portugal, and thus brucellosis is still a zoonotic health problem in these countries [12]. Besides, international travel and illegal trafficking of herds and dairy products through national borders are also factors that cannot be negligible.

**Figure 1:**
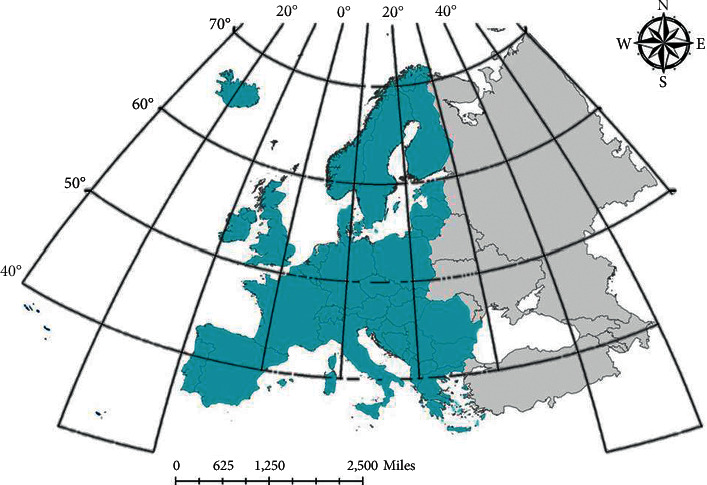
Twenty-five countries in Europe of the study area.

In Europe, case data are less affected by statistical bias and untimely treatment. At the same time, European countries have a small area and close communication, which can more fully reflect the epidemic characteristics of infectious diseases and facilitate spatial analysis and prediction. Therefore, this paper selects major European countries as the research object to analyze and predict the distribution of brucellosis. According to the data of the European Center for Disease Control and prevention, the final study area is determined as 25 major countries in Europe, and the specific scope is shown in Figure 1.

### 2.2. Data Introduction

The data selected in this study are mainly divided into the following parts.

#### 2.2.1. Case Data

The case data of brucellosis is the core data of this study. There are two main sources: the database of the World Animal Health Information System (OIE-WAHIS) of the World Organization for Animal Health and the report of zoonotic infectious diseases of the European Center for Disease Prevention and Control (ECDC).

OIE-WAHIS is a unique comprehensive database. The database reports and disseminates information about animal health around the world. OIE-WAHIS data reflect the information collected by the veterinary service departments of OIE member and nonmember countries and regions on livestock and wildlife diseases, emerging diseases, and zoonosis listed in OIE. OIE-WAHIS provides the number of cases of brucellosis of different strains through its data analysis platform. However, due to the reported number and its main focus on animal diseases, there is a certain lack of data.

ECDC is an EU institution to strengthen Europe's defense against infectious diseases. Its core functions include paying attention to epidemic information, providing corresponding report data, giving prevention suggestions, etc. It provides case data of major European countries in the annual report of zoonotic infectious diseases, including the number of diseases, gender, and age distribution.

This paper takes the data provided by ECDC as the main case data, and the disease situation provided by OIE-WAHIS as the auxiliary case data. The final data include the number of cases in 2008–2018 main countries in Europe (annual and monthly), incidence rate, gender, and age distribution.

#### 2.2.2. Vector Data of European Region

In order to carry out the research of spatial analysis and ConvLSTM prediction model, this research needs space vector data in Europe, and the source is OpenStreetMap (https://www.openstreetmap.org). Select European data through the official website of OSM, and you can download the SHP vector file of European national administrative divisions.

#### 2.2.3. Impact Factor Data

In order to make a better prediction, this study needs to collect data on other influencing factors that may be related to the incidence of brucellosis. According to the analysis of epidemic characteristics, the influencing factors mainly include socioeconomic factors, animal husbandry, and food. The data comes from the FAO statistical database (FAOSTAT, https://www.fao.org/faostat/zh/#home) of the Food and Agriculture Organization of the United Nations (FAO).

The FAO statistical database provides free food and agriculture data for more than 245 countries and regions, covering all data available to all FAO Regional Groups since 1961 to the latest. By selecting the classification required in FAOSTAT, we can obtain the corresponding data, select the corresponding classification, and finally obtain the impact factors of all European countries.

Due to different data sources and different formats and names, sorting and cleaning are needed to ensure the smooth progress of the study. The data sorting of this study takes the case data of major European countries of ECDC as the core to sort and clean the data.

After data cleaning and processing, we get the influencing factors of brucellosis in various countries from 2008 to 2018, including the following categories: cattle stock, sheep stock, butter supply, cheese supply, skimmed milk supply, beef consumption, mutton consumption, grassland and pasture area, population, and GDP.

Considering that the subsequent model will adopt the gradient descent algorithm for corresponding training, and the eigenvalue ranges between different influence factors are different, and there is a large gap, it is also necessary to store a normalized data sample for subsequent research and accelerate the convergence speed of the model. Based on this, we adopt the min-max normalization method, and its formula is as

(1) $$\begin{array}{c} x^{*}=\frac{\text{initial value}-\min\text{ value}}{\text{max value}-\min\text{ value}}. \end{array}$$

## 3. Method

### 3.1. Grid Feature Engineering

In this paper, we choose European countries as the research scale. Because there are few case data of brucellosis, the case data of brucellosis are directly mapped in the vector layer, and the results cannot fully reflect the spatial proximity attributes of adjacent countries. Therefore, in order to highlight the local spatial relationship brought by convolution layer, on the premise of keeping the topological relationship between countries unchanged, this paper divides the research area into grids.

**Figure 2:**
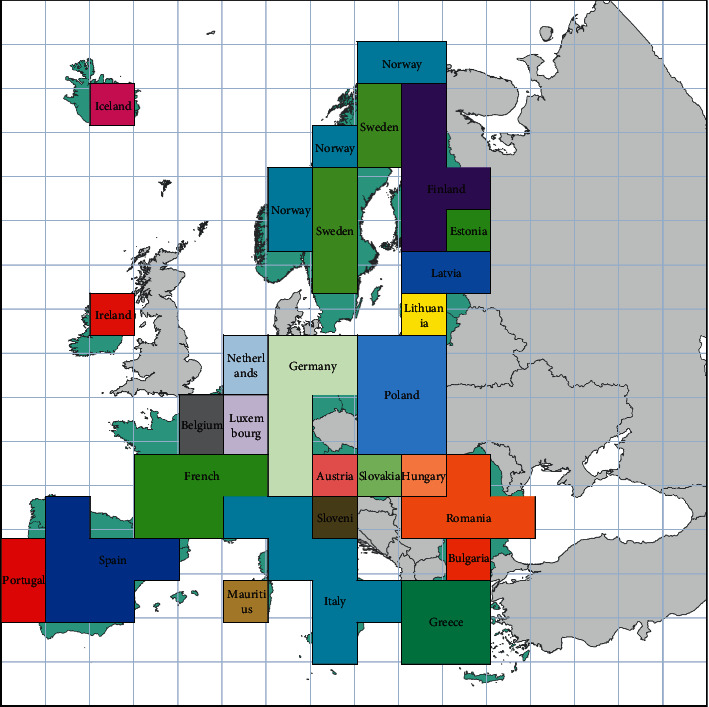
The grid is divided according to the area of the country.

We take the spatial region of the longitude and latitude under the European Terrestrial Reference System and map the observed values in the data set to a spatial region limited by longitude and latitude through 16*∗*16 grid division. When meshing, we start from the country with the smallest area and retrieve the grid of the country one by one. If the area exceeds one-third of the grid, it is marked as the national grid. On the contrary, the size of each grid is compared. If one grid occupies more than twice the area of others, the grid is selected as the country mark. If it does not exist, we will select the grid with the smallest area occupied by other countries in the grid occupied by that country as the marker. After processing, the results are shown in Figure 2.

Different countries use different grid representations. We divide the case data of different countries by the number of grids to obtain the specific data of each grid. To reduce skewness, we logarithmically convert the counts in pixels and normalize them on the scale of 0–255, and the gray value of the image represents the specific value.

### 3.2. The GeoGrid-Based ConvLSTM Network

**Figure 3:**
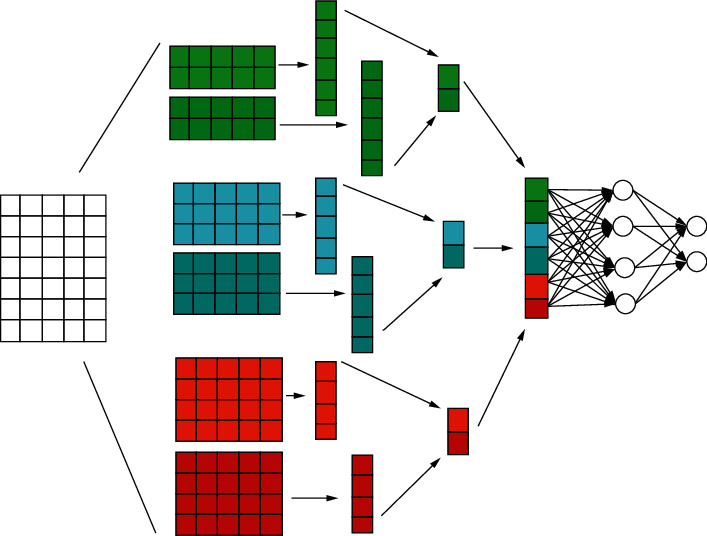
ConvLSTM network structure diagram.

ConvLSTM recurrent neural network can combine the spatiotemporal information attached to the constructed three-dimensional tensor (multidimensional influence factor vector) for spatiotemporal prediction [13, 14]. The overall structure of the model consists of five parts: input layer, Conv layer, pooling layer, LSTM layer, full connection layer, and output layer. Firstly, all the information to be input is preprocessed to obtain a vector with spatial and local features that can be input in the convolution, and then input it into the convolution layer to start training. The vector in the convolution layer can be used as a filter to extract local features to enhance the spatial performance of the model. Then, the information from the conv layer is input into the LSTM layer for training. This layer can represent the timing characteristics, reflect the influence of time, and finally reach the output layer through the full connection layer [15]. Through the gradient descent training method, the data of previous years are trained, and finally the future data are predicted. For the model structure, appropriate parameters need to be selected according to needs to achieve the best training effect.  Figure 3 is the algorithm flow chart of ConvLSTM.

Next, we will analyze the structure of each layer and the corresponding parameter selection in detail.

### 3.3. Activation Function

In the neural network, each level needs to use the activated function to unify and measure it. At the same time, it also enhances the learning ability of complex physical features. In the training, the activated function can calculate the weight error, and the neural network is optimized through the reverse propagation mechanism. In this paper, *tanh function* is selected as the activation function of neural network model.

*Tanh function* is a hyperbolic function, which can map the input value between (−1, 1) to realize the unification of measurement. The implementation method is shown in

(2) $$\begin{array}{c} f\left(x\right)=\frac{e^{x}-e^{-x}}{e^{x}+e^{-x}}, \end{array}$$

(3) $$\begin{array}{c} f^{\prime }\left(x\right)=1-\tan\;h^{2}x. \end{array}$$

#### 3.3.1. Conv Layer

The Conv layer in the neural network is composed of several Conv units. Each Conv unit can optimize the training data through the back-propagation algorithm. The purpose of Conv operation is to extract the input features. The Conv layer is connected with the top full connection layer, and multiple Conv layers can extract different features iteratively. The parameters of Conv layer include Conv core size, stride, boundary expansion, and input and output channels.

The model selected in this experiment includes a Conv layer. The filter is used to perform Conv operation and extract local features. Assuming that the size of the sliding window is *h*, the size of the filter is *h∗k*. The filter operates on each sliding window. The method is shown in

(4) $$\begin{array}{c} u\left(i\right)=f\left(w\cdot x_{i:\left(i+h-1\right)}+b\right), \end{array}$$

where *u*(*i*) represents the Conv output value of a filter at the position *i* and *f*(·)is a nonlinear activation function.

#### 3.3.2. ConvLSTM Network Prediction Model Training

As an extension of LSTM, ConvLSTM adds a conv layer before the general timing prediction LSTM, so that it can have a Conv structure in the state transition from input to and from transmission to. The specific method is as

(5) $$\begin{array}{c} i_{t}=\sigma \left(W_{xi}*x_{t}+W_{hi}*H_{t-1}+W_{ci}\circ C_{t-1}+b_{i}\right),\\ f_{t}=\sigma \left(W_{xf}*x_{t}+W_{hf}*H_{t-1}+W_{cf}\circ C_{t-1}+b_{f}\right),\\ C_{t}=f_{t}\circ C_{t-1}+i_{t}\circ \;\tanh\left(W_{xc}*x_{t}+W_{hc}*H_{t-1}+b_{f}\right),\\ O_{t}=\sigma \left(W_{xo}*x_{t}+W_{ho}*H_{t-1}+W_{co}\circ C_{t-1}+b_{o}\right),\\ H_{t}=O_{t}\circ \;\tanh\left(C_{t}\right). \end{array}$$

The method of combining conv neural network and LSTM model is used, and a *softmax layer* is added at the top of the model again. This method is used to train the whole model by minimizing the error of cross entropy. Given a training sample *X*^(*i*)^, its real label *y*^(*i*)^ ∈ [0,1] and the estimated probability *y*^(*i*)^ ∈ [0,1], where *j* ∈ [1,2,…*k*]. The error is defined as shown in

(6) $$\begin{array}{c} L\left(X^{\left(i\right)},y^{\left(i\right)}\right)=\sum_{j=0}^{1}1\left\{y^{\left(i\right)}=j\right\}\log\left({\tilde{y_{j}}}^{\left(i\right)}\right). \end{array}$$

When {·} is true, {*y*^(*i*)^=*j*} is 1; otherwise, it is 0.

## 4. Result

### 4.1. Prediction Result

According to the method described above, the data at each time, including the vector layer of cases and ten layers of influence factors, are used as an input layer to train the vector data of brucellosis. According to the relevant conclusions of the trend characteristics in Section 3, the prediction in this section is divided into two parts, which are called ConvLSTM (1) and ConvLSTM (4) for training and prediction of monthly data and stage data (one stage every four months). This experiment is carried out in Google cloud.

**Figure 4:**
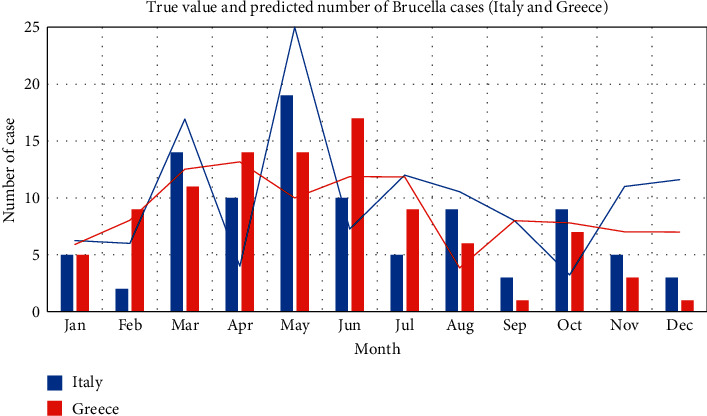
High incidence rate ConvLSTM model monthly forecast results chart (Italy and Greece).

**Figure 5:**
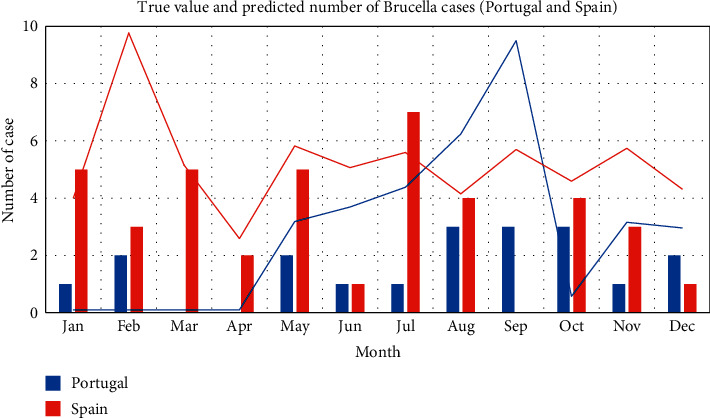
High incidence rate ConvLSTM model monthly forecast results chart (Portugal and Spain).

First, training the monthly data: taking a total of 120 data in each month from 2008 to 2017 as the training set, the brucellosis case data in each month of 2018 are predicted. Among them, the case prediction in high incidence rate countries is shown in Figure 4 and Figure 5. In the figure, the bar chart represents the real data, and the line chart represents the predicted value.

It can be seen from the figure that the prediction effect of monthly data has been significantly improved compared with LSTM, especially that the overfitting effect of the later section of the overall curve is optimized compared with LSTM. However, it can also be seen that the prediction effect of the model for the first few months is generally better than that for the following months. In other words, the prediction effect of the model for cases is weakened over time.

**Figure 6:**
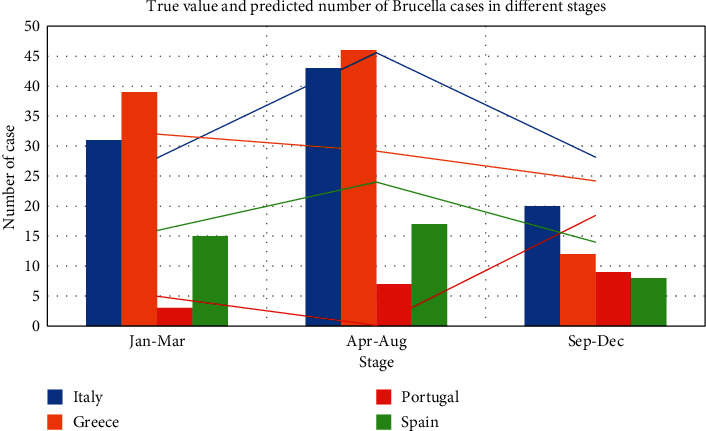
High incidence rate ConvLSTM model forecast results chart (every 4 months).

To deal with the problem that the prediction effect of the model on cases will be weakened over time, we sum the data every four months, compile them into stage data, and input them into ConvLSTM network. The results of the high incidence rate of brucellosis are shown in Figure 6. In the figure, the bar chart represents the real data, and the line chart represents the predicted value.

After comparison, we can find that the stage prediction results of ConvLSTM model are better than those of monthly prediction and LSTM model at the same time. It can also be seen from the prediction results that there are certain stage characteristics in the epidemic of brucellosis, and the prediction model can also reflect this characteristic. At the same time, the results of this section can also prove that ConvLSTM has a good effect on the prediction of brucellosis in major European countries with local spatial attributes.

### 4.2. Model Comparison

**Figure 7:**
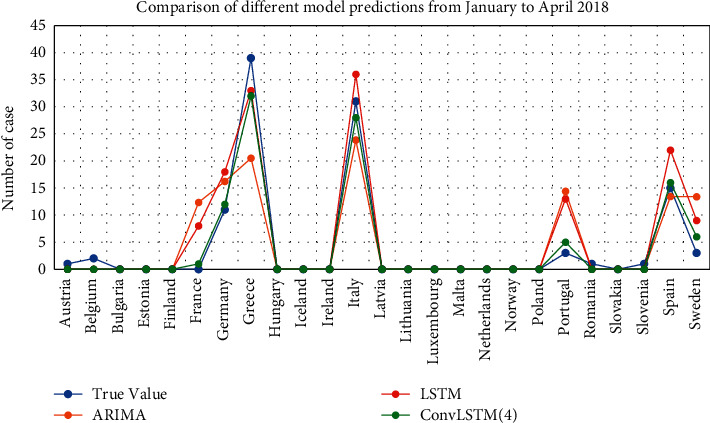
Comparison of different model predictions from January to April 2018.

In this paper, the results of brucellosis case data in 2018 obtained from LSTM, ConvLSTM monthly data prediction, and ConvLSTM stage data prediction are summed to obtain the case prediction from January to April 2018. The results of the prediction of the number of cases in Europe are shown in Figure 7.

**Figure 8:**
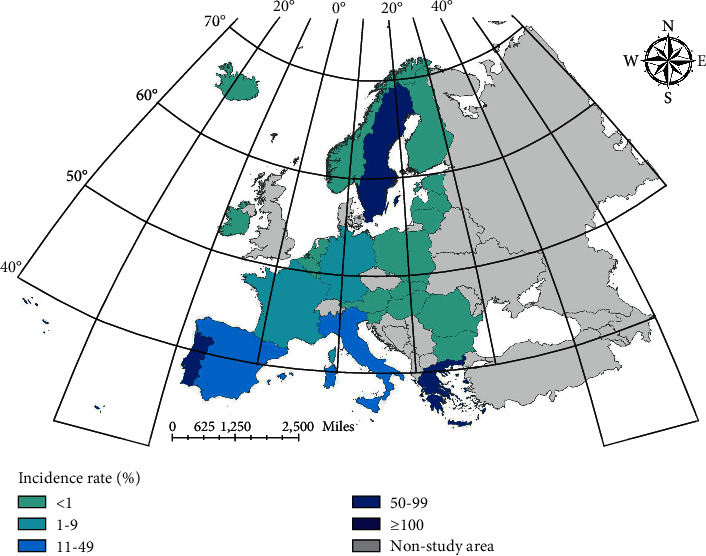
True value distribution of Brucella cases.

**Figure 9:**
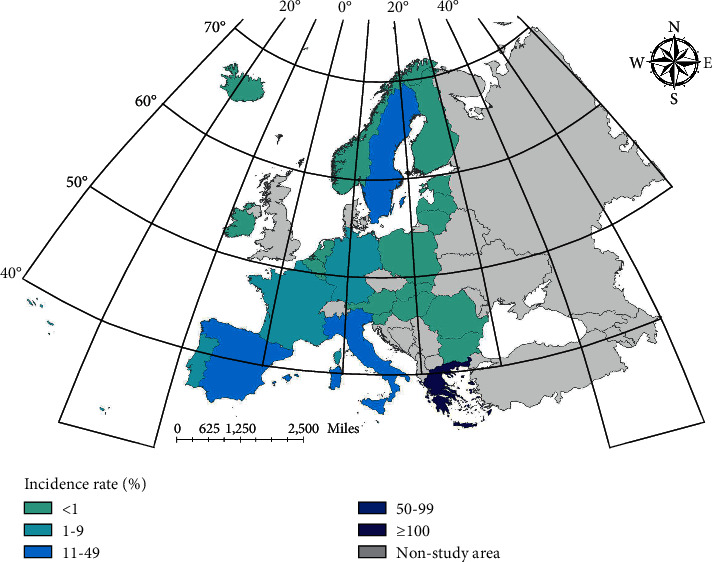
Spatial distribution of prediction data (ARIMA).

**Figure 10:**
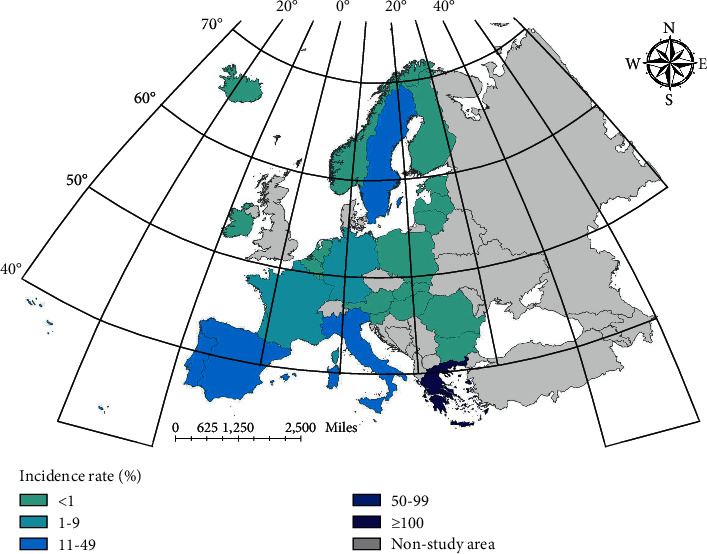
Spatial distribution of prediction data (LSTM).

**Figure 11:**
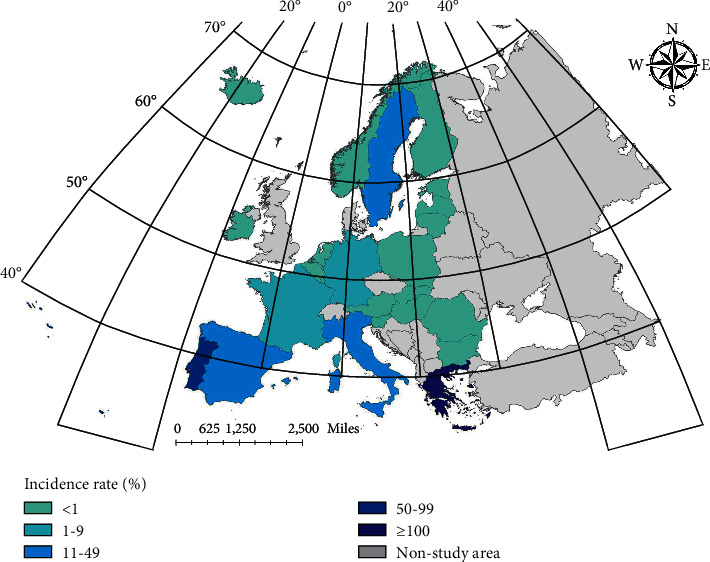
Spatial distribution of prediction data (CovnLSTM).

Combining the results of ARIMA, we can conclude that the prediction results of incidence rate of LSTM and ConvLSTM are better than that of ARIMA model. By adding Conv layer, vectorizing the data and using ConvLSTM model for prediction, the prediction effect will be higher than that of LSTM model. In the first mock exam, the prediction of brucellosis can be achieved better by using the epidemic trend stage.  Figure 8 shows the spatial distribution of real cases. Figures 9–11 show the spatial distribution of predicted case data of different models.

In this paper, the evaluation method of brucellosis prediction is established to predict the specific effect of the model and scientifically evaluate the experimental results. For the effect of the model, the statistical method is adopted to calculate the deviation between the fitted predicted value and the real data, and the root mean square error (RMSE) is used to determine the degree of fitting. The lower the RMSE, the smaller the estimation deviation between the estimated predicted value of the fitted prediction model and the real data and the higher the degree of fitting. In the limited measurement times, the specific calculation method of root mean square error is shown in the following equation:

(7) $$\begin{array}{c} \text{RMSE}=\sqrt{\frac{1}{N}\sum_{i=0}^{N}\left|x_{i}-\bar{x_{i}}\right|}. \end{array}$$

In the above formula, *N* is the time measurement (year or month) of the fitted data and $\left|x_{i}-\bar{x_{i}}\right|$ is the deviation between a group of measured values and the true value.

Due to the better short-term prediction effect of some models, we uniformly calculate the prediction root mean square error from January to April 2018. The error results of ARIMA's single country time series prediction, LSTM model's single country time series prediction, ConvLSTM monthly data prediction, and ConvLSTM stage data prediction are shown in Table 1.

From the error calculation results, it can be seen that the prediction effect of LSTM and ConvLSTM models is better than ARIMA model. The results of ConvLSTM model are better than LSTM. The ConvLSTM model using stage data is better than the ConvLSTM model using monthly data. This result is consistent with our previous conclusion.

## 5. Discussion

The purpose of our study is to explore the epidemic characteristics and spatiotemporal distribution of *Brucella* by comparing the spatiotemporal sequence prediction methods of *Brucella* and we collected, preprocessed, and applied the multisource data. In the stage of spatiotemporal sequence analysis, the visualization methods of temporal data and spatial data are used. In the model prediction stage, ARIMA time series prediction model, LSTM cyclic neural network model, and ConvLSTM model are constructed. In the result evaluation stage, RMSE is used to evaluate the result accuracy.

The results showed that the brucellosis prevalence in major European countries was periodic, which differed among countries. The incidence of brucellosis in Italy and Greece peaked in May and June, while in Spain it peaked in September. A possible explanation for this might be that the seasonality of brucellosis in Italy was related to the availability of dairy products at high risk for contamination in the spring, and in Greece it was linked to the peak period of slaughter and parturitions among farm animals, especially during the Orthodox Easter Season [16, 17]. Compared with Italy and Greece, Spain and Portugal had lower incidence of brucellosis, and the inconsistency of seasonality may be due to demographic, occupational, and socioeconomic factors as more than half of the reported brucellosis cases in the Iberian Peninsula lived in cities [18].

Through the comparison of the first mock exam results, the prediction results of LSTM and ConvLSTM models are obviously better than those of ARIMA models. The prediction results of ConvLSTM and Conv models are better than those of LSTM models by using the ConvLSTM layer model and the data of vectorization. Although ConvLSTM has a good effect on the prediction of brucellosis in major European countries with local spatial attributes, compared with the model, the effect of the initial prediction is better, and the later prediction has unstable results, which may be due to the overfitting of the subsequent prediction of Conv neural network. In addition, with the extension of prediction time, the instability of prediction itself is also one of the possible reasons [19]. The follow-up research hopes to further improve the prediction results, such as optimizing the convolution layer, adopting higher-level network, improving parameter and mechanism learning, and improving the prediction stability.

## 6. Conclusions

The core research work of this paper focuses on the spatiotemporal feature extraction and development trend model prediction of human brucellosis in major European countries. The prediction results have shown that LSTM and ConvLSTM models have higher forecast precision and could be further improved with divided trend stages. The major European countries with high prevalence of brucellosis include Greece, Italy, Portugal, and Spain, among which Greece was at increased risk and instability of human brucellosis infection. The findings of this study contribute to our understanding of the application of machine learning in epidemic disease prediction and provide effective decision support and theoretical basis for the prevention and control of *Brucella* in relevant departments.

## Figures and Tables

**Table 1:** Root mean square error of different models.

ARIMA	LSTM	ConvLSTM
5.73	3.83	1.73

## Data Availability

The data that support the findings of this study are available on request from the corresponding author. The data are not publicly available due to privacy restrictions.

## Abbreviations

ConvLSTM: Convolutional long short-term memory
LSTM: Long short-term memory
Conv: Convolutional
ARIMA: Autoregressive integrated moving average
RMSE: Root mean square error
GDP: Gross domestic product
OSM: Open Street Map
ECDC: European center for disease prevention and control
FAO: Food and Agriculture Organization of the United Nations
WAHIS: World Animal Health Information System.
